# Surgical Conscience

**Published:** 1908-12

**Authors:** R. M. Harbin

**Affiliations:** Rome, Ga.


### Journal-Record of Medicine

**Successor to Atlanta Medical and Surgical Journal, Established 1855, and Southern Medical Record, Established 1870.**

**OWNED BY THE ATLANTA MEDICAL JOURNAL CO.**

**Published Monthly.**

*Official Organ Fulton County Medical Society, State Examining Board, Presbyterian Hospital, Etc.*

EDGAR G. BALLENGER, M. D., Editor.
BERNARD WOLFF, M. D., Supervising Editor.
A. W. STIRLING, M. D., C. M., D. P. H., J. S. HURT, B. Ph., M. D., Associate Editors
E. W. ALLEN, Business Manager.

COLLABORATORS

DR. W. F. WESTMORLAND, General Surgery.
F. W. McRAE, M. D., Abdominal Surgery.
H. F. HARRIS, M. D., Pathology and Bacteriology.
E. B. BLOCK, M. D., Diseases of the Nervous System.
MICHEL HOKE, M. D., Orthopedic Surgery.
CYRUS W. STRICKLER, M. D., Legal Medicine and Medical Legislation.
E. C. DAVIS, A. B., M. D., Obstetrics.
E. G. JONES, A. B., M. D., Gynecology.
R. T. DORSEY, Jr., B. S. M. D., Medicine.
L. M. GAINES, A. B., M. D., Internal Medicine.
J. N. LeCONTE, M. D., Diseases of the Stomach and Intestines
L. B. CLARKE, M. D., Pediatrics.
EDGAR PAULIN, M. D., Opsonic Medicine.
R. R. DALY, M. D., Medical Society.
A. W. STIRLING, M. D., Etc., Diseases of the Eye, Ear, Nose and Throat.
BERNARD WOLFF, M. D., Diseases of the Skin.
E. G. BALLENGER, M. D., Diseases of the Genito Urinary Organs.

VOL. X. DECEMBER, 1908. No. 9

### SURGICAL CONSCIENCE.*

BY R. M. HARBIN, M. D., ROME, GA.

In his poem entitled "Latter Day Warnings," Dr. Oliver Wendell Holmes facetiously predicted a millenium:

"When legislators keep the law,
When preachers tell all they think,
When lawyers take what they would give.
And doctors give what they would take,
Then order your ascension robe."

Were he living today he might add another line: "And surgeons cut only when they would be cut."

While medicine has advanced to the position of a science, at the same time the ethical side of the profession has made corresponding progress and I am optimistic enough to believe so far

*Read before the Floyd County Medical Society, Rome, Ga., October 10, 1908.

as the doctor is concerned, that the millenium was not as far away as the physician poet dreamed and that there are many surgeons of the present day who would not do unto others what they would not have done unto themselves.

Yet in our ambitious zeal we are prone to wander away from the golden rule standard and all of us need further advancement in the ethics of our profession.

Billroth once wrote, "Years and experience bring in their train a certain degree of hesitancy," for conservatism is begotten of experience. In all scientific endeavor, strange as it may seem, conservatism is among the last accomplishments—one that is of necessity thrust upon every earnest student of truth. While we are pitying the lack of knowledge of our surgical forefathers, we should not forget that our posterity may pity us in the same way. More progress has been won in surgery in the last twenty five years than had been gained in twenty-five centuries and may it not be that our present attainment is only a beginning?

Surgeons like all true scientists, are earnest seekers of the truth. The truth of the advisability of any operation in question can only be revealed by the subsequent history of the patient operated on, but by painstaking investigation of all the circumstances and a careful review of the histories of previous cases, the result can be foretold with almost positive assurance by a conservative judgment. Of course aberrant cases may arise when no man can predict and such possibilities might be incidentally referred to. Judgment must be broader than zeal and conservatism more prevalent than radicalism. Because operations are radical, gives no guarantee of cure, unless it is clearly proven that a diseased condition of the extirpated organ was a cause of the complaint.

There are cases on the border line when operation is a question of election, such as gall stones, gastric ulcer, uterine fibroids, hernia, etc. We should carefully weigh all reasons for or against operation, and explain and submit to the patient for final approval. If the patient should decline, deal charitably with him, and do not threaten him with sudden death from a ruptured gall bladder, for he may live his allotted term of years. While we are considering the wrongs of radicalism, we must not forget the evils of conservatism. Timid conscience has put many patients beyond the reach of surgical treatment; more especially is this true of cancer. Our fear of alarming the patient and our hopes of a mistaken diagnosis will rob the patient of the only chance of recovery from an incipient cancer of the breast, for this is the only treatment known to be of value.

We can not but admire the zeal of the specialist. This zeal on the one hand and the ignorance of the patient on the other, often bring about a certain amount of disappointment to both parties.

It is inspiring to the patient to be told by the oculist that a cause of the inveterate headaches has been found which evaded the family physician. The oculist conscientiously believes his diagnosis, but will the subsequent history prove it? The patient meets the same disappointment with the gastrologist and finally consults a surgeon who wonders that an incarcerated ovary had not been thought of, and advises ovariotomy. The surgeon firmly believes his diagnosis, and proceeds with the treatment. The artificial climacteric produces unexpected results and by this time the patient has left the arena of scientific medicine only to adopt some neuro-fad. The patient will excuse a tentative diagnosis in all classes of specialists except surgery and do we not justify the patient?

It may be asked how shall we proceed? After availing himself of all possible knowledge of the patient, the surgeon should explain the probable results, then let the patient or family share the responsibility of what ever decision that may follow and a positive declaration of assent should be secured. It is wise to reduce technical knowledge to the layman's comprehension, then a common sense consultant from the family should become a party to the decision.

We are occasionally asked if we would guarantee a cure from a certain operation. It imght be well to reply to such questions that a human life in apparent health cannot be guaranteed under any circumstances. The surgeon should explain the probabilities as he understands them.

My teacher, Dr. J. D. Bryant once remarked to his class that it was not so dangerous to take ether as it was to cross Broadway down town, and it would be well to explain the dangers of an operation by some sort of comparison like that. However, simple an operation may be, we can estimate the degree of danger, but we should never say there is absolutely no danger in any operation.

It seems strange that anaesthetics have received so little attention until recently as a contributing cause of danger in surgery and I do not know but that I had rather be a patient in the hands of a careless surgeon than a careless anaesthetist. In uncomplicated major operations, probably one of the most common contributing causes of shock arises from an unusual susceptibility and over-dosage of the anaesthetic. Using the average amount may be overdosage for some individuals may be a law unto themselves. I recall two uncomplicated cases of this class sustaining shock. One was a case of double tubo-ovarian cyst and the other a pan-hysterectomy for fibroids, the latter having been fatal in twelve hours. Allowing the patient to show resistance occasionally is only a slight inconvenience to the operator and lessens the dangers of a tedious operation.

Some of us conclude at times that it is our duty to operate when the patient comes with a decision already formed. Only recently a woman applied for an examination for fibroid tumors of the uterus and was disappointed when extirpation was not advised because little inconvenience was experienced and she was approaching the menopause. The question of operation on apparently hopeless patients is a difficult one. Surgery is often thus brought into disrepute among some who are even very intelligent.

It may be considered a duty by some surgeons to satisfy patients by saying there was a catarrhal inflammation of the appendix or a cystic ovary when apparently normal organs were removed. Technical language cannot continuously conceal facts from intelligent laymen.

Of course there are difficulties of diagnosis in abdominal surgery that are as yet insurmountable. No diagnostic table can differentiate satisfactorily between gastro-intestinal toxaemia and a fulminating appendicitis within the first few hours and according to the present teachings many normal appendices have been removed, because of the impossibility of diagnosis. An occasional needless operation is no stigma on surgery when many times more lives are saved.

In every profession a certain amount of error is unavoidable and surgery is no exception but it is gratifying that there is being made marked progress in eliminating error.

Operative measures intend to relieve supposed diseased conditions are and should be in themselves practically harmless and no surgeon should ever advise surgical treatment when the operation is as much or more dangerous than the disease itself. These questions are the most difficult and require the most careful deliberation that is ever exacted of the surgeon. The late Dr. Flint was wont to say to his students: "If you can't do your patient any good be sure you don't do any harm" and this injunction would apply to ministers and lawyers as well as physicions and surgeons. No surgeon should advise a laparotomy to establish a diagnosis when he would not be willing to accept the same treatment and all exploratory operations should be explained and it must be remembered that the zeal of the patient is not perhaps so excessive as that of the surgeon in settling certain surgical questions. While we are paying allegiance to the science of medicine we must not forget the interest of the patient for these two interests do exceptionally conflict. We would not wish to prove the hazard of some vaunted procedure on our patients even though it would thus contribute to scientific medicine. An ambitious surgeon may feel that he can never win fame by confining his work to the orthodox teachings of surgery. Be that as it may, the environment of private practice does not warrant such a policy. I once advised the attempt to aspirate the lateral ventricles through a trephined opening after failing to locate a probable abscess of the brain with hemiplegia following grip, the patient having been in an apparently hopeless condition. The experimental nature of the operation was explained. The procedure seemed to relieve and a rapid recovery followed. A fecal impaction with a nucleus of cheese in the caput coli once led me to advise laporatomy for appendical abscess. The patient declined to submit and in a few days the impaction was discharged. When a surgeon makes dogmatic assertions he may become a victim of chagrin. It is not the duty of the surgeon to persuade any sane and intelligent patient into operation. All he should do is to point out the necessity and emphasize the dangers for no surgeon can afford to take the risk of any operation done under forced persuasion.

Note worthy evidence of ethical advancement is marked in the abandonment of an old prejudice that the calling of a consultant is a reflection of the skill of the attending surgeon. It is gratifying that we now find so few such instances of lack of education. Honest difference of opinions will be productive of good so long as truth and the patient's wellfare are subserved, but when any ulterior, selfish motives arise, consultations miscarry. The truth of surgical questions is not written in black and white and even a life long study does not eliminate all errors. Sir Frederick Treves once said that any of his assistants could carry out the technique of his surgical work, but his long experience had not enabled him to always say whether, when or how he should operate.

The safest judgment is not necessarily associated with positive opinions and self opinion may be the cause of many abortive brilliant careers in surgery. Dividing opinion with our confreres is occasionally a method of learning that we unconsciously gain in attempting to be generous.

I am glad to state that I have never heard of the pernicous custom of fee-splitting in our state. Whenever any underhand policy is resorted to, it is only a question of time when the facts will crop out and the results will be embarrassing.

We are often tempted to over charge a grateful patient with ordinary means who has undergone an operation that unquestionably saved a life. The patient is amply grateful and feels that he is paying for his life and at the time may be willing to give a good per cent, of his possessions. An exorbitant fee may be satisfactory for the time being, but later he will become dissatisfied and feel that he had been wronged. The patients ability to pay requires consideration as well as the surgeons skill. A physician's personal necessities should never have any influence in determining the question of charges and for that reason he should practice economy. On the other hand a moral deliquent may be ungrateful for meritorious service and the physician should become one member of society to attempt to correct such defects so far as a tactful policy will allow. A patient may justly feel that he should not pay as much for an operation that fails to relieve as the one that accomplishes its aim, although the same skill was bestowed in both instances and under such circumstances the surgeon hould make some concessions. A dissatisfied patient is a menace to the pleasure as well as the success of a surgeon's or physician's work. The free gift of professional services should never be made as such, even to real objects of charity for it tends to crush the remnant efforts at self sustenance for as a rule pay patients are more grateful than those who never expect any attempts at renumeration. The physician will lend moral support to a charity patient by assuring him that any attempts towards paying will be appreciated and the transaction should never close by making it a free gift. When any one who is financially able accepts without pay, conscientious professional services, which are universally admitted to be worthy of renumeration, he is guilty of robbing the family of the physician or surgeon and it is a duty after explaining the reasonableness of the fee to insist on payment even threatening legal measures, although suing is a doubtful propriety and should be made a last resort. Perhaps the wiser course would be to decline future calls. In fact, the physician is morally reprehensible for failing to attempt collecting reasonable fees for he encourages the beneficiary to get something of recognized value for nothing, to say nothing of the injustice done the other capable, conscientious and energetic members of the profession who depend on their work for a livelihood and a patient who acquiesces in such a transaction deteriorates his sense of honor and becomes less likely to appreciate meritorious services. Pay patients usually monopolize the time of the man who never sends a bill and for that reason he may do less real charity than others. The query arises, would not a capable physician or surgeon accomplish more real charity by collecting his bills and donate a share of his earnings to some good cause. The medical man as the member of a society is expected to pay his debts and in the interest of his patients to devote his entire time to his profession and it would be grossly inconsistent for him not to collect his fees for the laborer is worthy of his hire. Illness as a rule, is no excuse for poverty in the patient for disease is one of the misfortunes of life that requires to be provided for and a man is not expected to earn that money to pay the expense thereof while thus afflicted and anyone who has not provided for such a contingency should be given the opportunity to make amends when restored to health.

Some useful careers in the medical profession have had melancholy endings from failure in paying due regard to the matter of collections. I don't know of anything more pitiable than a physician, who has spent a useful career without preparation for a rainy day and when the infirmities of age circumscribe his activities, he is made to feel that the necessities of the case compel a greater activity. His weaknesses tend to embitter him toward his life long patients who without due regard to past obligations elect to choose younger and more active members of the profession. Perhaps many of you have heard of a notable exception in the late Dr. Rhett, of Charleston, who was one of the best known surgeons in the South. Having repeatedly heard that something like $50,000 was raised by popular subscriptions among his friends and patients for his family, I wrote Dr. Lane Mullaly of that city to ascertain the facts. He wrote me that Dr. Rhett was never known to send a bill and that only $6,000 or $7,000 was raised principally by those who felt they owed him, for the support of his widow and seven children. Such instances of the donation of conscience money in the medical world are rare and in this instance the amount paid was a mere pittance for doubtless any year of his active life could earn double that amount.

For some unknown psychological reason the matter of collection of fees is repugnant to a great many capable medical men and requires to be overcome as a duty to ourselves, as a debt to our families, and in justice to our patients and profession.

